# Potential Use of Andean Tuber Waste for the Generation of Environmentally Sustainable Bioelectricity

**DOI:** 10.3390/molecules29091978

**Published:** 2024-04-25

**Authors:** Segundo Rojas-Flores, Magaly De La Cruz-Noriega, Luis Cabanillas-Chirinos, Nélida Milly Otiniano, Nancy Soto-Deza, Nicole Terrones-Rodriguez, Mayra De La Cruz-Cerquin

**Affiliations:** 1 Institutos y Centros de Investigación de la Universidad Cesar Vallejo, Universidad Cesar Vallejo, Trujillo 13001, Peru; mdelacruzn@ucv.edu.pe (M.D.L.C.-N.); notiniano@ucv.edu.pe (N.M.O.); nsoto@ucv.edu.pe (N.S.-D.); nterronesr@ucv.edu.pe (N.T.-R.); mdelacruz@ucv.edu.pe (M.D.L.C.-C.); 2 Investigación Formativa e Integridad Científica, Universidad César Vallejo, Trujillo 13001, Peru; lcabanillas@ucv.edu.pe

**Keywords:** sustainability, organic waste, Olluco, bioenergy, bioelectricity

## Abstract

The growing demand for agricultural products has increased exponentially, causing their waste to increase and become a problem for society. Searching for sustainable solutions for organic waste management is increasingly urgent. This research focuses on considering the waste of an Andean tuber, such as Olluco, as a fuel source for generating electricity and becoming a potential sustainable energy source for companies dedicated to this area. This research used Olluco waste as fuel in single-chamber microbial fuel cells using carbon and zinc electrodes. An electric current and electric potential of 6.4 ± 0.4 mA and 0.99 ± 0.09 V were generated, operating with an electrical conductivity of 142.3 ± 6.1 mS/cm and a pH of 7.1 ± 0.2. It was possible to obtain a 94% decrease in COD and an internal resistance of 24.9 ± 2.8 Ω. The power density found was 373.8 ± 28.8 mW/cm^2^ and the current density was 4.96 A/cm^2^. On day 14, the cells were connected in earnest, achieving a power of 2.92 V and generating enough current to light an LED light bulb, thus demonstrating the potential that Olluco waste has to be used as fuel in microbial fuel cells.

## 1. Introduction

The continuous increase in the world’s population has caused the demand for the production and distribution of different types of food to grow exponentially in recent years [1]. From 2000 to 2020, the generation of primary foods increased by 50%, which generated a solid economic boost for the agricultural sector [2]. This has led to increased waste generated in the distribution chain until consumption increases. In developing countries that do not have an organized system for its collection, they are heavily contaminated due to the dumping of this waste in unsuitable places [3].

On the other hand, remote places where autochthonous fruits, vegetables, or tubers are planted and harvested do not have adequate electricity supply [4]. In 2018, it was reported that 77% of the population had an energy supply; that is, 23% (1671 million people) still needed an electricity supply [5]. Due to this, many cities with this problem have decided to generate and obtain other sustainable and renewable energy [6]. For example, in 1990, no one used bioenergy as a source to obtain electricity; however, in 2022, there are reports that it produced 675.11 TWh of electrical energy according to the Energy Institute’s Statistical Review of World Energy (2023) [7].

These two problems can be solved through microbial fuel cell (MFC) technology, which uses waste with organic content as fuel. It works through the conversion of chemical energy into electrical energy through the oxidation-reduction process [8]. MFCs have been investigated using waste from fruits, vegetables, and tubers; for example, Yaakop et al. (2023) used domestic organic waste as fuel, managing to generate voltage peaks and a power density of 110 mV and 0.1047 mW/m^2^ [9]. Likewise, Ahmad A. (2023) also used potato waste as a substrate in his single-chamber MFCs, managing to generate 150 mV with an internal resistance of 724 Ω and a power density of 1.450 mW/m^2^ [10]. Kitchen waste has also been used, generating a power density of 0.76 W/m^3^ and registering an optimal operating pH of 5.74 [11]. The chemical–biological compatibility that the electrodes used must be optimal because the electrical energy is obtained from the metabolic activity of the microorganisms present in the substrate [12]; the microorganisms release electrons and these are captured by the anode electrode and travel through an external circuit to the cathode electrode. This has led to the use of different types of electrodes, the most economical being those that have obtained the best voltage, current, and power density: metallic nature electrodes [13]. Although MFCs currently have a series of difficulties scaling up, they are presented as a long-term alternative. Because the components they need for their operation are expensive, studies like these that provide alternatives to improve are vital.

In Peru, the production of Olluco (*Ullucus tuberosus*) has increased; this Andean tuber has been used for hundreds of years for gastronomy, and in recent decades due to investments in agriculture, it has begun to be produced in large quantities for export [14]. Currently, it has been reported that they have produced more than 60,000 tons in 2023, becoming one of the agricultural products with the greatest export potential. Olluco contains vitamins (A, B, and C), calcium, iron, phosphorus, a high fiber content, and a low percentage of sugar; in addition, betalains, betaxanthins, and betacyanins are Olluco’s most predominant compounds [15]. The literature does not report on the use of Ullucus tuberous waste as fuel in microbial fuel cells. This research is the first report on this technology using this type of substrate.

The main objective of this research is to observe the potential of Olluco as a fuel source in single-chamber microbial fuel cells using carbon electrodes. For this, the pH, voltage, electrical conductivity, electrical current, and chemical oxygen demand (COD) values were observed for 28 days. The internal resistance of the MFC and the power density as a function of the current density were also observed, and the identification of the microorganisms on the anode electrode took place. In this way, a new and innovative way of using Olluco waste will arise, using it as fuel in MFCs for electricity generation, which could help illuminate remote places, those lacking this supply in an environmentally sustainable way.

## 2. Materials and Methods

### 2.1. Construction of the Single-Chamber Microbial Fuel Cells

**Figure 1:**
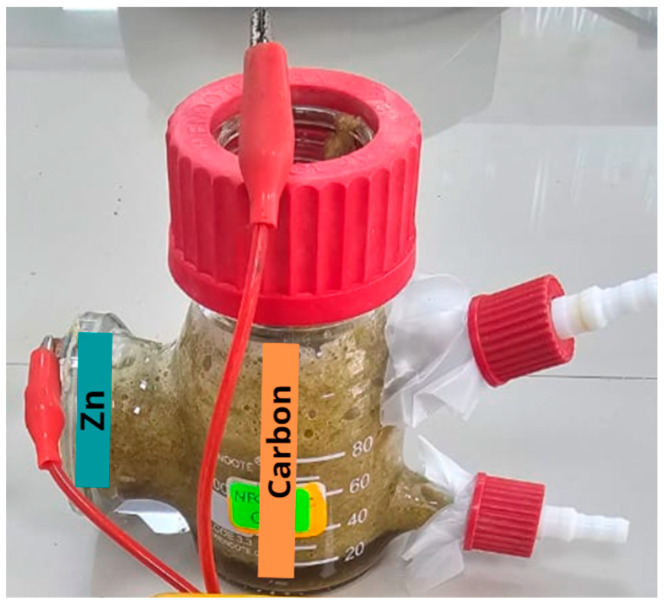
MFC design used.

The single-chamber microbial fuel cell was obtained from the Xin Tester company (Shanghai, China), which was made of silica (SiO_2_). The electrodes were made of carbon (anode) and zinc (cathode) as reported by Aguero et al. (2023) [16], and a proton exchange membrane/Nafion 117 (Wilmington, DE, USA) was used. The anodic electrode’s area was 30 cm^2^, and the cathodic electrode’s area was 15.75 cm^2^; both electrodes were joined by an external circuit whose resistance was 100 Ω (see Figure 1).

### 2.2. Collection of Samples Used as a Substrate

The substrate used was collected from the waste of the Santo Domingo Market, Trujillo, La Libertad, Peru, whose samples were selected by the same sellers and then taken to the laboratory of the Cesar Vallejo University, collecting 1.5 kg, which was washed several times until all types of impurities acquired from the environment were eliminated. Then, it was left to dry at 25 °C for 48 h; finally, with an extractor (extractor, Labtron, LDO-B10-Orlando, FL, USA), a 550 mL liquid solution was obtained. This solution was used as a substrate and finally used in the microbial fuel cells. The initial parameters are shown in Table 1.

### 2.3. Characterization of the Electrochemical Parameters of the Microbial Fuel Cells

The voltage and electrical current values were monitored for 28 days using a digital multimeter (Truper MUT—830 Digital Multimeter, Truper, Campeche, Mexico) and an external resistance of 100 Ω. The COD (chemical oxygen demand) values were measured using the closed reflux colorimetric method according to the NTP 360.502:2016 standard [16]. The internal resistance values of the MFC were carried out using an energy sensor (Vernier ±30 V and ±1000 mA). The PD (power density) and CD (current density) values were obtained with Nafion 117 using the formula PD = V^2^_cell_/(R_ext._A) and CD = V_cell_/(R_ext._A) where the V_cell_ is the voltage of 118 and the MFC and Rext are 0.3 (±0.1), 3 (±0.6), 10 (±1.3), 50 (±8.7), 100 (±9.3), 220 (±13), 460 (±23.1), 119 531 (±26.8), 700 (±40.5), and 1000 (±50.6) Ω [16]. Three MFCs were carried out, where each MFC contained 100 mL of crushed Olluco waste (Olluco residues). The average value and standard deviation were calculated from the results obtained.

### 2.4. Isolation and Molecular Identification of Electrogenic Bacteria

The bacteria were isolated from the anodic chamber using the swabbing technique to recover the most significant number of bacteria. Using the streaking technique, the sample contained in the swab was sown on the surface of the Petri dishes with Nutrient Agar and Mac Conkey agar [17]. The seeded plates were incubated at 35 °C × 24 h; after the incubation time, the growth of colonies was observed. Colonies with different cultural characteristics (color, shape, and the macroscopic appearance of the colony) were then replicated in slant tubes with nutrient agar.

Before performing molecular identification, Gram staining was performed to verify the purity of the strains. The already-numbered axenic cultures were sent to the external laboratory ECOBOTECH LAB SAC for molecular identification through 16S rRNA gene sequencing.

## 3. Results and Analysis

**Figure 2:**
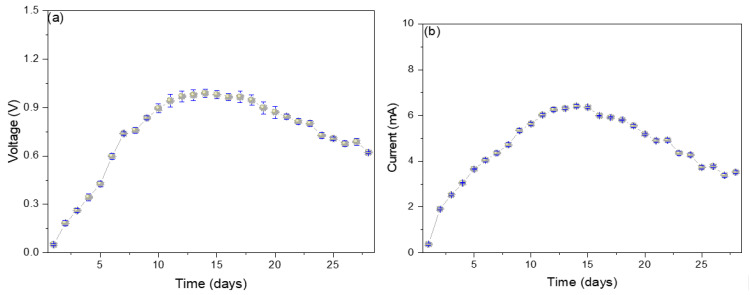
Monitoring values of (**a**) voltage and (**b**) electric current of reverse microbial fuel cells.

Figure 2a shows the electrical potential values increase from the 1st day (0.05 ± 0.01 V) to the 14th day (0.99 ± 0.09 V) and then slowly decay until the last day (0.62 ± 0.14 V). The progressive increase in the electrical potential shown is due to the potential differential originating between the electrodes in the oxidation-reduction process that occurs within the MFC. Over time, the components necessary to initiate the oxidation-reduction process begin to decrease, which are the values shown on the monitoring days [18]. It has been reported that the use of glucose in the substrate stimulates the increase in the electrical potential; it has been possible to increase from 66.6 mV at 2.78 mM of glucose to 96.4 mV at 5.56 mM because foods with glucose concentration will consume various competitive metabolisms (methanogenesis and fermentation) by inhibiting other components of the substrate [19]. In their research, Kalagbor et al. (2020) managed to generate voltage peaks of 4.2, 3.1, and 3.0 V using tomato, banana, and pineapple waste as a substrate in single-chamber MFCs, mentioning that the decrease in potential values is due to the reduction in the organic matter available in the substrate [20]. Kebaili et al. (2021), in their double-chamber MFCs, used fruit waste leachates as a substrate, managing to generate peaks of 260 mV, mentioning that the growth of sucrose in the former allowed high voltage values, while the accumulation of metabolic waste produced the decrease in the electric potential [21]. Likewise, Rahman et al. (2021), in their single-chamber MFC, used orange, banana, and mango waste as substrates, managing to generate peaks of 259, 255, and 320 mV, mentioning that the substrates that contained a more significant amount of glucose were those that showed higher voltages [22]. The electric current values increased from the 1st day (0.40 ± 0.05 mA) to the 14th day (6.4 ± 0.4 mA) and then decreased until the 28th day (3.5 ± 0.6 mA). The microbes present in each substrate act as biocatalysts, stimulating the degradation of the organic matter present, thus generating electrons, which are captured by the anode electrode and travel through the external circuit to the cathode electrode, thus generating electricity [23]. Yahoob et al. (2022) used local waste (rambutan, langsat, and mango) from Malaysia, managing to generate 0.026 mA using mango as a substrate, attributing the decrease in electrical values to the decrease in carbon in the substrate [24]. Blueberry waste has also been used as substrates in single-chamber MFCs, managing to generate peaks of 1.130 ± 0.018 mA, attributing the rapid increase in electrical values to the high presence of dissolved oxygen and the good formation of the anodic biofilm responsible for capturing the electrons generated in the medium [25]. Naveenkumar M. and Senthilkumar K. (2021) used tannery wastewater to generate peaks of 0.43 mA, attributing the high electron release values to the low resistances shown by the MFC [26]. The existence of a relationship between the mechanisms and the degradation pathways of organic matter is revealed by electrogenic microbes that use two types of metabolisms (anaerobic and fermentative), where one uses anaerobic respiration and the other produces primary redox metabolites. The fermentative metabolism generates small organic acids and alcohols, which are used in anaerobic respiration. In contrast, the sugars that can be found in the substrate can be used up in the fermentation process as in methanogenesis [11,19,23].

**Figure 3:**
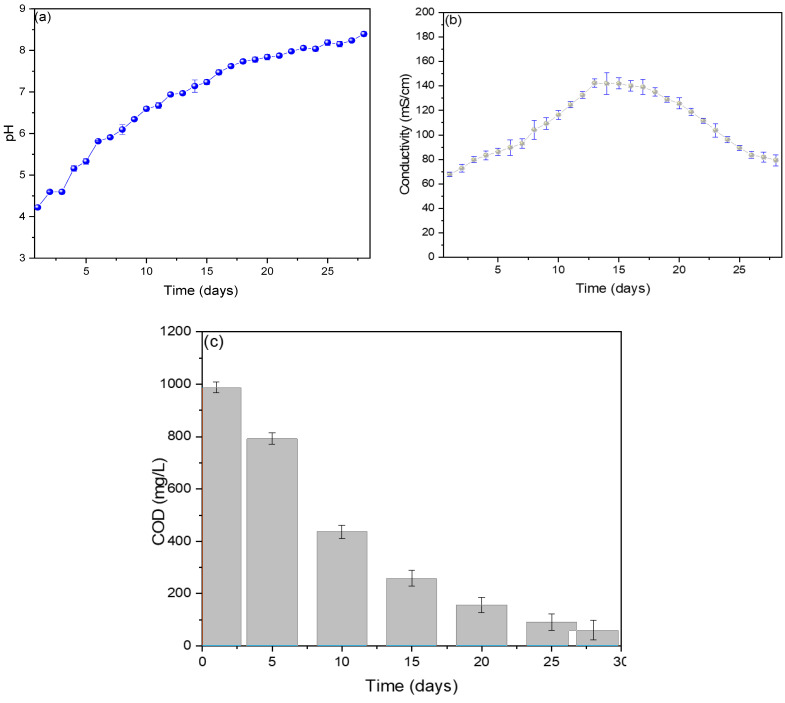
Monitoring of (**a**) pH, (**b**) conductivity, and (**c**) COD values of reverse microbial fuel cells.

The pH values are shown in Figure 3a, observing an increase in values from the first (4.2) to the last (8.4 ± 0.6) day, with the optimal operating pH of the MFC of 7.1 ± 0.2. The generation of electric current depends on the different mechanics that microbes have for the generation of electrons due to their different forms, and these microbes grow at specific pH values, which is why the standardization of an optimal pH in each MFC is important [27]. Rincon et al. (2022) standardized its optimal operating pH at 7.2, using banana waste as substrates in their single-chamber MFC, managing to generate voltage and electric current peaks of 300 mV and 0.2867 mA, attributing the pH value to the state of maturity of the banana used and its components (oxalic, citric, and malic) [28]. Molasses has been used as a substrate in single-chamber MFCs, achieving an optimal pH of 5.25 ± 0.12, and it was shown that the increase in pH value causes the performance of the MFC to decrease due to the variation in the ionization state of the amino and carboxyl functional groups that are present in the bacterial biofilm of the anode electrode [29]. Verma M. and Mishra V. (2023) used banana peel as fuel in their MFC, obtaining an optimal pH of 7.4, showing a maximum voltage and current of 600 mV and 0.30 mA [30].

Figure 3b shows that the electrical conductivity values increased from the 1st day (67.8 ± 1.7 mS/cm) to the 13th day (142.3 ± 6.1 mS/cm) and then decreased slightly until the 28th day (79.3 ± 8.8 mS/cm). The ions released in the first days due to oxidation-reduction reactions caused the substrate to increase its conductivity. In contrast, the sedimentation of organic compounds in the last few days is responsible for decreased electrical conductivity [31]. Similar behaviors have been reported in other investigations, mentioning that the variations in electrical conductivity depend on the internal resistance of the MFCs, which vary due to the behavior of the microorganisms in generating electric current [32]. The measured COD values are shown in Figure 3c, where a 94% decrease can be observed on the last day (60.3 ± 36.5 mg/L) from its initial value (987.5 ± 10.3 mg/L), showing a decrease of 74% for the 14th day (258.2 ± 30.6 mg/L), which was when the maximum peaks of voltage and electrical current were observed. It has been shown that there is a relationship between the decrease in COD and the values of the electric current; this is due to the activity of electron-producing microbes, which are diminished by the consumption of the organic charge in the substrate [33]. For example, Din et al. (2020) used potato waste as substrates in their single-chamber MFCs, observing that the degradation rate is higher than the hydrolysis rate, which could be due to the active oxidation of the substrate by the microorganisms present in the substrate [34]. Toczyłowska-Mamińska R. and Mamiński M. (2023) mention in their research that the aeration of the cathode chamber increases COD reduction and power density [35].

**Figure 4:**
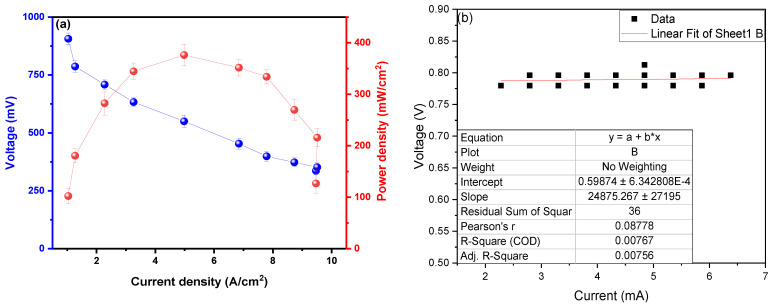
Values of (**a**) power density as a function of current density and (**b**) internal resistance.

The maximum power density observed was 373.8 ± 28.8 mW/cm^2^ at a current density of 4.96 A/cm^2^, with a peak voltage of 901.1 ± 19.8 mV, as shown in Figure 4a. Din et al. (2023) managed to generate a PD_max_ of 0.3714 mW/cm^2^ for a CD of 0.235 mA/cm^2^ using potato waste as fuel, deducing that the formation of the thickness of the anodic biofilm affects the PD values; the biofilm is influenced by the temperature at which the MFC operates [36]. Du H. and Shao Z. (2022) used potato peels and activated sludge as substrates, generating PD_max_ peaks at 14.1 mW/m^2^ for a CD of 320.1 mA/m^2^, deducing that increasing hydrolysis rates improves the performance of the redox reactions of the soluble fractions present and promote electron transport, improving PD values [37]. Radeef A. and Ismail Z. (2021) used waste from the potato chip processing industry as a substrate in their MFCs, observing a PD_max_ of 434.8 mW/m^2^ in the CD of 1165.6 mA/m, mentioning that the distance between the electrodes interferes with the results of the PD, as well as the size and material of each electrode used in the MFC [38]. The use of metallic electrodes facilitates the passage of electrons through the electrode area and between the chambers (anodic and cathodic) due to the high inherent electrical conductivity of the material [39], which explains the high power density values observed in this investigation. Similarly, Sonawane et al. (2024) developed their microbial fuel cells using organic waste, successfully generating 0.17 V and 400 mA/m_2_ of electrical potential and maximum power density; also, a 60% reduction in COD was observed, and it was possible to reduce total solids by 82.83% [11]. Ohm’s law (V = RI) is used in Figure 4b to calculate the internal resistance of the MFC; for this, the values of the electric potential in the “*Y*” axis and in “*X*” were adjusted values of the electric current, whose slope of the linear fit is the internal resistance of the MFC. The internal resistance (R_int_) calculated was 24.9 ± 2.8 Ω; this low value would demonstrate the high voltage, electric current, electrical conductivity, and power density shown in this research. Choudhury et al. (2021) conclude that R_int._ values influence microbes’ metabolic processes, biofilm formation, and attachment to the electrode [40]. Seed et al. (2022) used leachate from landfills and municipal wastewater as a substrate in their MFC, mentioning the R_int_ values. They increase when there are poor degradation kinetics of organic matter, causing ohmic resistance and anodic depletion to increase [41]. Tamilarasan et al. (2024) calculate an R_int._ of 95.257 Ω using petrochemical effluents as substrates, mentioning that the anode electrode, electrolyte, and membrane influence microbial adhesion and electron transfer and that materials with a high surface area can reduce internal resistance by promoting efficient electron transport [42]. Lawson et al. (2020) managed to calculate an R_int._ of 62 ± 4 Ω in their MFCs using ferricyanide as substrate, mentioning that any O_2_ diffusion in the analyte does not affect the resistance of the anode [43]. The extraction of the genetic material and the sequencing were performed using the software MEGA (Version 4.0, Molecular Evolutionary Genetics Analysis), shown in Table 1.

In Table 2, we can see the identified microorganisms, which were two strains of *Stenotrophomonas maltophilia* with identity percentages of 99.32 and 99.59%. The results obtained are similar to those obtained by other authors, who found this microorganism as part of a microbial consortium that generated maximum peaks of 4.459 ± 0.0608 mA and 0.991 ± 0.02 V for current and voltage in onion residues [17]. In addition, *S. maltophilia* is cosmopolitan because it can be found in a wide variety of habitats, including the *rhizosphere* of crop soils [44]. On the other hand, it has been reported that the *S. maltophilia* bacteria form an anodic biofilm (above the biofilm) with a good structure of extracellular polymeric substances (EPSs), which protects antimicrobials and foreign agents whose inhibitory agents are the protein bifunctional phosphoglucomutase/phosphomannose y glucose-1-phosphate [45]. Likewise, Shu Hui Liu et al. (2022) reported that *S. maltophilia* could remove copper and generate bioelectricity in microbial fuel cells using sediments with high concentrations of copper [46]. Meanwhile, Gnanarathinam et al. (2023) found *S. maltophilia* to be part of a microbial consortium that has the property of reducing Cr^+6^ to Cr^+3^ using a dual-chamber microbial fuel cell (MFC) [47]. Alvarado-Gutierrez et al. (2020) demonstrated the biotechnological capacity of *S. maltophilia* to degrade the fungicide carbendazim, considered an ecotoxic contaminant that is frequently found in water reservoirs [48]. 

**Figure 5:**
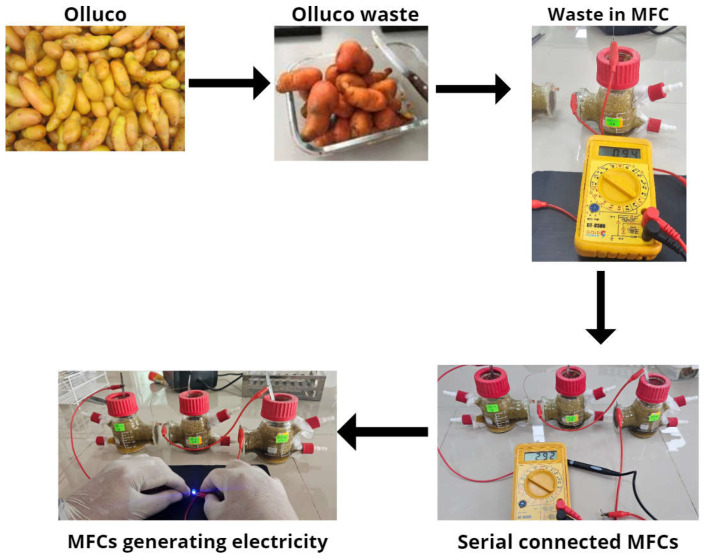
Schematization of the electrical energy generation process through waste.

The Olluco waste is seen in Figure 5, where the crushed waste was placed in single-chamber microbial fuel cells, and when connected in series, they managed to generate an electrical potential of 2.92 V on the 14th day, enough to ignite an LED spotlight, demonstrating that the waste of this tuber from the Andes can have great potential to be used as fuel and generate electricity. Although these electrical values still need to be higher to apply to larger scales, the voltage and current values found in this research are quite high, which would not give hope due to the values and costs reported by other research [49]. It has been reported that for the treatment of 113.14 m^3^ of waste, there is a cost of USD 4.2 million [50]. The treatment of 1 m^3^ of organic waste costs around USD 289, and the expense is more significant in the manufacture of electrodes [51]. Another way to improve the economic viability of microbial fuel cells is by using the sediments generated after waste treatment and the generation of electrical energy as fertilizer or some other type of application [52]. It has also been reported that the use of microbial fuel cells as bioremediations or for the reduction of heavy metals are other applications that can be given to this technology, and it has already been shown that the excess concentration of heavy metals reduces the growth of microbes in cells [53]. The optimal concentration level of metals would also have to be standardized to improve the performance of the systems by reducing maintenance and operating costs and time [54].

In recent years, research on the use of different organic wastes has increased abruptly because the agroindustry has begun to produce large quantities of waste and has the need to reuse their waste efficiently and profitably. Table 3 shows recent research on waste in microbial fuel cells for bioelectricity production compared to our research. In the comparison of results, it can be observed that electrodes of a metallic nature or with metallic components significantly increase power density and current density compared to those that use only carbon, graphite, or some other derivative. This shows that our research follows the trend of using metal electrodes combined with carbon to take advantage of the porosity of the latter. Furthermore, it has been observed that the use of MFCs connected in series to increase the output voltage of the MFCs has started to gain importance; for example, Rokhim et al. (2024) used four MFCs connected in series, generating a maximum voltage of 5.53 V [55]. Likewise, using bianodes in MFCs has also been reported to improve the efficiency of the cells because by placing two anodes, the adhesion of electrons would increase, thus obtaining a greater flow of electrons. However, a problem has been observed in the reaction of these electrodes because the cathode tends to show faster wear compared to cells with a single anode [56,57]. Other researchers have reported that the coating of the metal electrode with carbon or graphite improves the adhesion of electrons because the porosity shown by these two materials adheres a more significant number of electrons than when passing to the metallic material if it repowers because the metal or non-metallic nanoparticles embedded in the carbon or graphite electrode show lower resistance to the passage of electrons [58,59].

## 4. Conclusions

This research successfully demonstrated that Olluco waste can be used as fuel in single-chamber MFCs using carbon and zinc electrodes managing to generate peaks of electric potential and electric current of 0.99 ± 0.09 V and 6.4 ± 0.4 mA, which operated at an optimal pH of 7.1 ± 0.2 and whose maximum electrical conductivity was 142.3 ± 6.1 mS/cm, on the fourteenth day. In addition, the chemical oxygen demand was reduced by 94% in the 28 days of operation of the MFCs, while the internal resistance calculated was 24.9 ± 2.8 Ω. The maximum power density calculated was 373.8 ± 28.8 mW/cm^2^ and the current density was 4.96 A/cm^2^; in addition, the three microbial fuel cells used were connected in series on day 14, managing to generate a potential of 2.92 V, turning on an LED spotlight. This research demonstrates the potential that Olluco waste has to be used as fuel, as well as the solution for farmers who grow and harvest this tuber because the areas where this work is carried out usually lack electrical energy. For future work, it is recommended to carry out a technical study on the application of the circular economy using Olluco waste for better sustainable development from the point of view of environmental, social, and economic development to give a descriptive point of view for the future of the sustainable bioenergy area. Also, this prototype must be scaled up with a more significant amount of Olluco waste, increasing the size of the electrodes and standardizing the optimal operating pH to the value found in this research. Isolated microbes should also be used as biocatalysts to increase voltage, electrical current, and electrical potential values in microbial fuel cells. 

## Figures and Tables

**Table 1:** Initial parameters of the Olluco waste solution.

Parameters	Values
Temperature (°C)	21.5 ± 0.5
Electrical Conductivity (mS/cm)	67.8 ± 1.7
Dissolved Oxygen (mg/L)	198.2 ± 2.5
Total Dissolved Solids (mg/L)	485.7 ± 4.4
Chemical Oxygen Demand (mg/L)	987.5 ± 10.3
pH	4.2

**Table 2:** Electrogenic bacteria present in the MFC anode-containing Olluco residues.

Identified Species	pb	BLAST	
		Identity (%)	Accession Number
*Stenotrophomonas maltophilia*	1459	99.32%	NR_112030.1
*Stenotrophomonas maltophilia*	1458	99.59%	NR_041577.1

**Table 3:** Comparison of electrical parameters with other types of organic waste.

Substrate Type	Types of MFC	V_max._ (V)	PD (mW/m^2^)	CD (mA/m^2^)	Electrodes	Ref.
Rice, vegetable, and fruit wastes	Single-chamber	5.53	12.719	2.300	Cu and Mg	[55]
Mango peel extract	Single-chamber	0.102	0.099	31.57	Graphite and carbon	[56]
Banana peel waste	Dual-chamber	602 ± 1.5	2.2 ± 0.1	129.4 ± 1.0	Stainless steel and activated carbon	[29]
Sweet lemon peels	Single-chamber	102.5	1.100	31.569	Carbon and graphite	[57]
Coriander waste	Single-chamber	882 ± 154	3 04.33 ± 16.51	506	Copper and zinc	[58]
Oily kitchen waste	Single-chamber cubic	400.011	25.156	35.213	Carbon and graphite	[59]
Olluco waste	Single-chamber	0.99 ± 0.09	373.8 ± 28.8	4.96	Cu and Carbon	This research

## Data Availability

Data are contained within the article.
